# Morphophysiology and Hormonal Control of the Mammary Gland of the Bat *Artibeus lituratus* During Gestation and Lactation: Emphasis on Estradiol and Progesterone

**DOI:** 10.1002/jmor.70097

**Published:** 2025-10-13

**Authors:** Cornélio S. Santiago, Pollyana B. Pimentel, Emília M. Soares, Juliana F. Ferraz, Luiz H. A. Guerra, Carolina C. Souza, Rejane M. Góes, Eliana Morielle‐Versute, Sebastião R. Taboga, Mateus R. Beguelini

**Affiliations:** ^1^ Universidade Federal do Oeste da Bahia (UFOB) Center of Biological and Health Science Barreiras Brazil; ^2^ Universidade Estadual Paulista (UNESP) Instituto de Biociências, Letras e Ciências Exatas (IBILCE), Department of Biological Sciences, São José do Rio Preto São Paulo Brazil

**Keywords:** Chiroptera, hormonal regulation, pregnancy, reproduction

## Abstract

*Artibeus lituratus* is an important species of bat of the Phyllostomidae family. Despite its wide distribution, detailed studies on the mammary gland of this species are lacking. Therefore, this study aimed to evaluate the development, lactation, and hormonal regulation of the mammary gland of *A. lituratus* during different reproductive stages, with an emphasis on estradiol and progesterone signaling. Fifteen sexually mature adult females were collected, divided into three sample groups based on their reproductive status and subjected to anatomical, histological, morphometric, and immunohistochemical analyses. The results revealed that the mammary gland of *A. lituratus* exhibited a pattern very similar to that of other mammals. However, some notable differences were observed, including an epithelium composed of epithelial, basal, and myoepithelial cells, as well as continuous remodeling, branching, and renewal of the gland during lactation, accompanied by marked epithelial cell death. The branching of the mammary gland during advanced pregnancy appears to be stimulated by elevated serum estradiol levels, which promote ductal proliferation via ERα activation, and by increased progesterone levels, which, through PR activation and in conjunction with prolactin, stimulate side branching and alveolar development.

## Graphical abstract

The mammary gland of NON females of *Artibeus lituratus* was composed of few ducts interspersed within a rich stroma, and covered by adipose tissue. The branching of the mammary gland in AP females are triggered by stimuli from an increase in serum estradiol that, through ERα activation, stimulates the proliferation of the ducts, and an increase in serum progesterone that, through PR activation, in conjunction with prolactin, stimulates side branching and alveolar formation. (AP, advanced pregnancy; Arrows, mammary tubules; At, adipose tissue; L, lumen; LAC, lactating; NON, non‐reproductive; S, stroma)

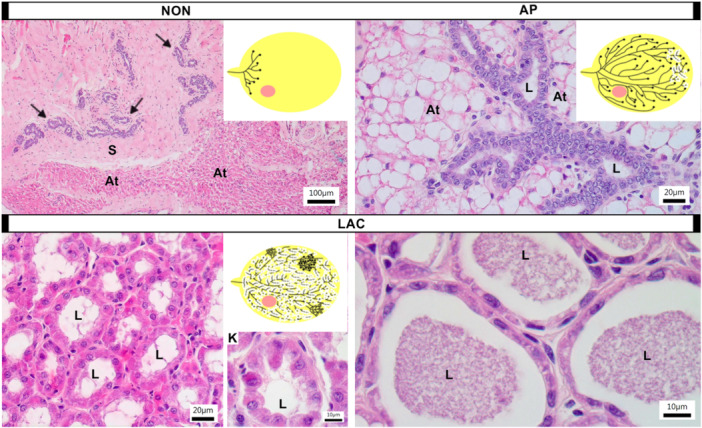

## 1 Introduction

Mammary glands are epidermal appendages that evolved from apocrine glands and exhibit a branched tubule‐alveolar morphology (Biswas et al. 2022). Their principal function is milk secretion (Biswas et al. 2022). During puberty, under hormonal stimulation from the pituitary and the ovaries, these glands grow and branch — the only organ with this capacity (Macias and Hinck 2012; Inman et al. 2015). During gestation, mammary glands develop and begin to secrete milk, a substance essential for newborn nutrition (Biswas et al. 2022). After the lactation, however, these glands tend to involute, especially in wild mammalian species (Macias and Hinck 2012).

Given the high susceptibility of human mammary glands to cancer — breast cancer being the second leading cause of death among women (Brisken and O'malley 2010) — the female mammary tissue has been extensively studied, particularly regarding its structure, development, and both normal and pathological function (Macias and Hinck 2012). In general, mammary gland development follows a similar pattern across most mammalian species. It begins during fetal life, with the formation of placodes and the emergence of a rudimentary ductal structure — events that occur prenatally. At puberty, under the influence of sex hormones, this rudimentary structure undergoes ductal elongation, branching morphogenesis, and the formation of lactiferous, intermediate, and terminal ducts. During pregnancy, the combined actions of progesterone and prolactin drive further branching and alveolar differentiation, resulting in a developed ductal tree. During lactation, the sucking stimulus of the newborn triggers the maturation of the gland and the secretion of milk (Biswas et al. 2022). After weaning, the absence of suckling initiates the involution process, wherein the gland is remodeled back to its pre‐pregnancy state (Biswas et al. 2022; Macias and Hinck 2012; Richert et al. 2000). The extent of involution varies among species — it is less pronounced in humans and cows, where glandular tissue remains evident, or more pronounced in wild species (seasonally breeders), such as bats, where the glands may nearly disappear (Crichton and Krutzsch 2000).

Despite these interesting characteristics, the mammary glands of bats remain poorly studied, especially in relation to their morphophysiology and hormonal regulation.

In recent decades, males of the bat *Artibeus lituratus* have been used as an experimental model in ecotoxicological studies, especially to assess the reproductive impacts of various pesticides (Amaral et al. 2012; Brinati et al. 2016; Machado‐Neves et al. 2018; Freitas et al. 2021; Oliveira et al. 2022). Similarly, recent works have shown that the female reproductive system of *A. lituratus* shares several similarities with humans, including an elongated, cylindrical, and simplex uterus; simple, unilateral, and non‐preferential ovulation; fundic and interstitial implantation; and a discoidal, hemochorial chorioallantoic placenta (Rodrigues et al. 2019). Additionally, this species exhibits similar hormonal fluctuations during pregnancy, with significant and gradual increases in estradiol and progesterone levels from early to late gestation (Santiago et al. 2020).

The selection of *A. lituratus* as an experimental model is not arbitrary. It is one of the best‐known species within the Phyllostomidae family, widely recognized for its adaptability to inhabit large urban areas and has broad geographic distribution, ranging from Mexico to northern Argentina (Reis et al. 2007). Although primarily frugivorous (Fleming 1986; Galetti and Morellato 1994; Passos and Graciolli 2004), *A. lituratus* can also consume floral parts, insects, and nectar (Sazima et al. 1994). Due to their capacity to fly long distances during foraging, these bats are important seed dispersers, contributing to habitat regeneration and playing a key role in ecological succession and restoration (Muller and Reis 1992; Passos and Passamani 2003).

Due to its great ecological importance, its use as an experimental model (Rodrigues et al. 2019; Santiago et al. 2020), and the marked involution of its mammary glands, a more in‐dept investigation into the organization, morphophysiology, and function of the mammary glands of *A. lituratus* is extremely important and urgent. Such research would provide valuable support for experimental studies and serve as a scientific foundation for the development of reproductive management strategies for this species.

Thus, the aim of this study was to characterize the morphology, branching, development, lactation, and hormonal regulation of the mammary glands of *A. lituratus* during its different reproductive phases (nonreproductive [NON], advanced pregnant [AP], and lactating [LAC]), and to evaluate how these glands are modulated by serum levels of estradiol and progesterone.

## 2 Materials and Methods

### 2.1 Capture and Licenses

All specimens were collected in the urban area of São José do Rio Preto, located in the northwest region of São Paulo State, Brazil, (49W22'45" 20S49'11"), between September 2013 and August 2014. Bats were captured at night using mist nets (3 × 6 m), strategically positioned to intercept bats flying 1–3 m above the ground. The nets were placed close to fruit trees, along potential flight routes or at the exits from shelters.

After removal from the nets, specimens were placed in individual cages (40 × 20 × 20 cm) and transported to the laboratory, where they were kept in a specific room in the dark at 25°C–30°C, with water provided ad libitum. On the morning following capture, the bats were euthanized, processed, and fixed.

The captures were authorized by the Brazilian Institute of Environment and Renewable Natural Resources (Instituto Brasileiro do Meio Ambiente e dos Recursos Naturais Renováveis – IBAMA – Process: 21707‐1). All experimental procedures complied with the Animal Research: Reporting of In Vivo Experiments (ARRIVE) guidelines; were conducted according to the National Institutes of Health Guide for the Care and Use of Laboratory Animals (NIH Publications N°. 8023, revised 1978), and approved by the animal experimentation ethics committee of the São Paulo State University (CEUA–UNESP, Process: 013/09). Access to Genetic Legacy was approved by the Genetic Legacy Management Council of the Brazilian Ministry of the Environment (Process: AA38F12).

### 2.2 Species, Experimental Design and Aging

This study focused on *A. lituratus*, a bat species that is not listed as endangered by the Red List of Threatened Species of the International Union for Conservation of Nature (IUCN).

Fifteen sexually mature adult females of *A. lituratus* were collected and divided into three sample groups (*n* = 5 per group) based on their reproductive status, according to Rodrigues et al. (2019): (a) nonreproductive (NON), specimens showing undeveloped nipples, minimal abdominal circumference, and absence of fetus; (b) advanced pregnancy (AP), specimens with undeveloped nipples, enlarge abdominal circumference, and the presence of a well‐developed fetus; and (c) lactating (LAC), specimens with developed nipples, reduced abdominal circumference, and absence of a fetus.

Since *A. lituratus* inhabits specific roosts with a harem social organization, composed of few specimens (8 to 16 individuals per colony), the capture of specimens can directly affect population structure and dynamics. Therefore, the number of specimens collected in this study was carefully determined as a balance between the minimum number required to perform statistically significant analyzes and the maximum number permitted by IBAMA to avoid disrupting colony integrity.

The adult females were identified based on body weight, the degree of wear of the coat and teeth, and the complete ossification of the metacarpophalangeal epiphyses (De Knegt et al. 2005; Rodrigues et al. 2019; Santiago et al. 2020; Beguelini et al. 2020, 2021).

The mammary glands of each specimen were subjected to histological, morphometric, and immunohistochemical analyses.

### 2.3 Processing of Animals

For standardization, on the morning following capture, the bats were euthanized by deep dissociative anesthesia, which consisted of a mixture of ketamine (370 mg/kg; Dopalen‐Vertebrands, Paulínia, SP, Brazil) and xylazine (16 mg/kg; Rompun‐Bayer S.A., São Paulo, SP, Brazil) applied at 0.1 mL per 50 g of body weight (0.08 mL ketamine plus 0.02 mL of xylazine). After confirmation of death, the mammary glands (right and left) were removed and processed for analysis.

Other materials were removed from all analyzed specimens for future molecular (lung, kidney, liver, and muscle) and reproductive (ovary, uterus, uterine tubes, and vagina) evaluations. After all materials had been removed, the bats were fixed in a 10% aqueous formaldehyde solution for 48 h, stored in a 70% aqueous alcohol solution, and deposited in the Scientific Chiroptera Collection of the Department of Zoology and Botany of IBILCE/UNESP (DBZ‐UNESP), current Department of Biological Sciences, where they will be available for taxonomic‐systematic studies.

### 2.4 Histology

The mammary glands were immersed in methanol, chloroform, and acetic acid (6:3:1) fixative solution for 3 h at 4°C; dehydrated in ethanol; cleared in xylene; and embedded in paraffin (Histosec ‐ MERK, Darmstadt, Germany). Histological Section (4 μm thick) were stained with hematoxylin and eosin – H&E, periodic acid and Schiff, alcian blue, Masson's trichrome, and Gomori's reticulin (Ribeiro and Lima 2000), or submitted to immunohistochemistry.

### 2.5 Morphometry

The mammary glands were evaluated to determine the relative percentage of the epithelium, lumen, and stroma (stereology). The analysis was performed using the Image‐Pro‐Plus software, version 6.0 (Media Cybernetics©) for Windows®. The data were collected by applying the M130 multipoint test system, according to Weibel (1963), with 130 points in the system. For this analysis, 20 fields of the H&E‐stained slides were randomly selected for each specimen. The relative frequency of each component was calculated according to the points of the test system that touch the analyzed component, that is, the epithelium, lumen, and stroma. Adipose tissue was considered to belong to the stroma.

### 2.6 Immunohistochemistry

Histological sections were deparaffinized, rehydrated, and submitted to antigen retrieval in citrate buffer (pH 6.0) at 92°C. Endogenous peroxidases were blocked using a solution of 10% hydrogen peroxide in methanol, and nonspecific antibody binding was blocked using 3% bovine serum albumin. The sections were incubated with primary antibodies against (Table 1) estrogen receptor α (ERα – rabbit polyclonal anti‐ERα, MyBioSource, Cat# MBS316681, RRID:AB_10577585), progesterone receptor (PR – mouse monoclonal anti‐PR (F‐4), Santa Cruz Biotechnology Cat# sc‐166169, RRID:AB_2166687), proliferating cell nuclear antigen (PCNA – mouse monoclonal anti‐PCNA, Santa Cruz Biotechnology Cat# sc‐56, RRID:AB_628110), activated caspase‐3 (mouse monoclonal anti‐Caspase‐3, Santa Cruz Biotechnology Cat# sc‐56053, RRID:AB_781826), the basal cell marker ‐ P63 (P63 – mouse monoclonal anti‐P63 [4A4], Santa Cruz Biotechnology Cat# sc‐8431, RRID:AB_628091), and α‐actin (mouse monoclonal anti‐α‐actin (1 A4), Santa Cruz Biotechnology Cat# sc‐32251, RRID:AB_262054). After primary antibody incubation, the sections were incubated with a specific polymer (Post Primary Block and Polymer, Novocastra, Newcastle Upon Tyne, UK, DAKO Envision™ + Duallink system‐HRP, K4061). The immunoreaction was visualized with diaminobenzidine (DAB, Sigma, St. Louis, MO), and the sections were counterstained with Harry's hematoxylin. Negative controls were performed the same way as described above, except the primary antibody was omitted. The reactions were performed in triplicate.

**Table 1:** Antibody table.

Antibodies	Source	Identifier
Rabbit polyclonal anti‐ERα	MyBioSource	Cat# MBS316681 RRID:AB_10577585
Mouse monoclonal anti‐PR	Santa Cruz Biotechnology	Cat# sc‐166169 RRID:AB_2166687
Mouse monoclonal anti‐ PCNA	Santa Cruz Biotechnology	Cat# sc‐56, RRID:AB_628110
Mouse monoclonal anti‐Caspase‐3	Santa Cruz Biotechnology	Cat# sc‐56053 RRID:AB_781826
Mouse monoclonal anti‐P63	Santa Cruz Biotechnology	Cat# sc‐8431 RRID:AB_628091
Mouse monoclonal anti‐α‐actin	Santa Cruz Biotechnology	Cat# sc‐32251 RRID:AB_262054

The relative percentage of ERα, PR, PCNA, and Caspase‐3 positive and negative cells in the epithelium and stroma was determined using measurement fields of the entire length of the slides.

### 2.7 Statistical Analysis

The data are expressed as the mean and standard deviation. All data were initially submitted to normality test (Shapiro‐Wilk test). Parametric data were submitted to one‐way analysis of variance (ANOVA) and, subsequently, to a Tukey test for multiple comparisons, whereas non‐parametric data were submitted initially to a Kruskal–Wallis test followed by Dunn's test. All analyses were performed using the Statistica 7.0 program (Statsoft^©^ Inc., Tulsa, USA). A *p* value of ≤ 0.05 was considered to indicate a statistically significant difference.

## 3 Results

### 3.1 Gross Anatomy

**Figure 1:**
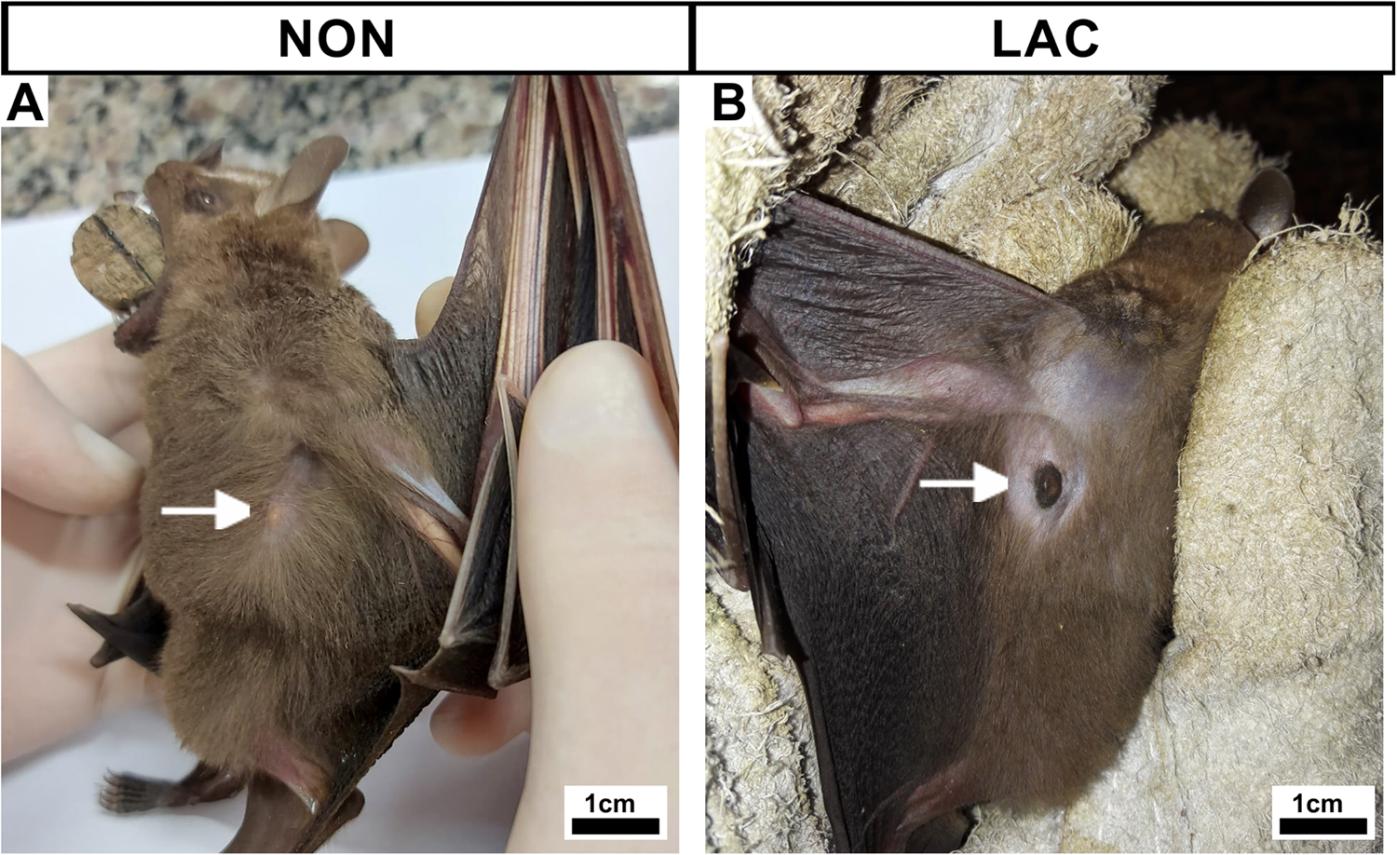
General pattern of the mammary gland of *Artibeus lituratus* in different reproductive phases. (A) Lateral view of a NON female. Mammary gland located in the axillary region, under the arm (white arrow), with small nipple covered with hair. (B) Lateral view of a LAC female. Well‐developed nipple with no surrounding hair (white arrow).

**Figure 2:**
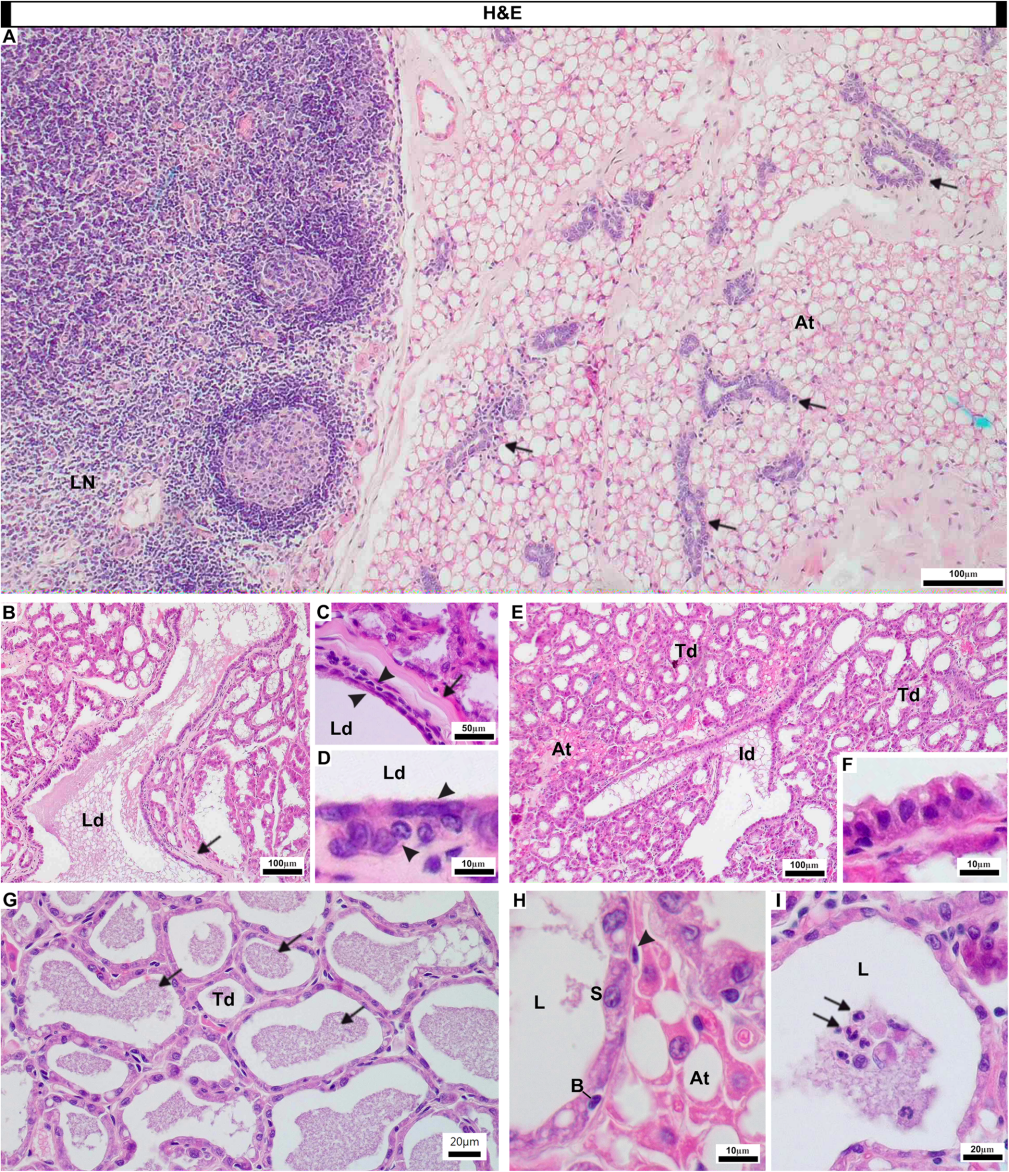
General pattern of the mammary gland of *Artibeus lituratus*. Histological sections stained with hematoxylin and eosin. (A) Overview of the mammary gland in an AP female. Close relation of the mammary gland to the axillary lymph node. Great amount of unilocular adipose tissue coating a ramified tubule‐alveolar gland (arrows), which branch into lobes that are not fully distinct. (B–D) Lactiferous ducts in LAC females. Lactiferous ducts are wide tubules surrounded by a thick sheath of connective tissue (arrows, B,C) and lined by an epithelium with a double layer of cuboidal cells (arrowheads, C,D). (E,F) Intralobular/intermediate ducts in LAC females. Intralobular ducts are smaller tubules that present epithelium with one or two layers of cells (F). (G–I) Terminal ductules in LAC females. Presence of milk in the lumen of the terminal ductules (arrows, G), which fully differentiate into secretory alveoli in LAC females. Secretory alveoli are lined by an epithelium (H) composed of cuboidal secretory cells, sparse basal cells and a compact outer layer of myoepithelial cells (arrowhead, H). Milk secreted in the lumen may present a certain amount of polymorphonuclear cells, mainly neutrophils (arrows, I). (At, adipose tissue; B, basal cell; Id, intralobular/intermediate duct; L, lumen; Ld, lactiferous duct; LN, lymph node; S, secretory cell; Td, terminal ductules).

*Artibeus lituratus* has a pair of mammary glands, located in the axillary region, under the arms (Figure 1A,B). Due to this anatomic positioning, the glands are typically in direct contact with the axillary lymph nodes (Figure 2A). Anatomically, NON and AP females exhibit small nipples, generally covered by hair (Figure 1A), whereas LAC females presents well‐developed nipples, usually devoid of surrounding hair (Figure 1B).

### 3.2 General Histology

The mammary glands of *A. lituratus* were surrounded by a variable amount of unilocular adipose tissue and exhibited a highly ramified tubule‐alveolar morphology, which branched into lobes that were not fully distinct (Figure 2A). The lactiferous ducts were wide tubules that originated at the nipples, surrounded by a thick sheath of connective tissue (Figure 2B,C), and lined by an epithelium with a double layer of cuboidal cells (Figure 2C,D). These lactiferous ducts branched throughout the gland and differentiated into smaller intralobular/intermediate ducts (Figure 2E), which presented epithelium with one or two layers of cells (Figure 2F). The intralobular ducts branched throughout the entire gland, immersed in adipose tissue, differentiating at their ends into terminal ductules (Figure 2E). During lactation, these terminal ductules differentiate into secretory alveoli responsible for milk production (Figure 2G). The alveoli were lined by an epithelium composed of cuboidal secretory cells, scattered basal cells, and a compact outer layer of myoepithelial cells (Figure 2H).

**Figure 3:**
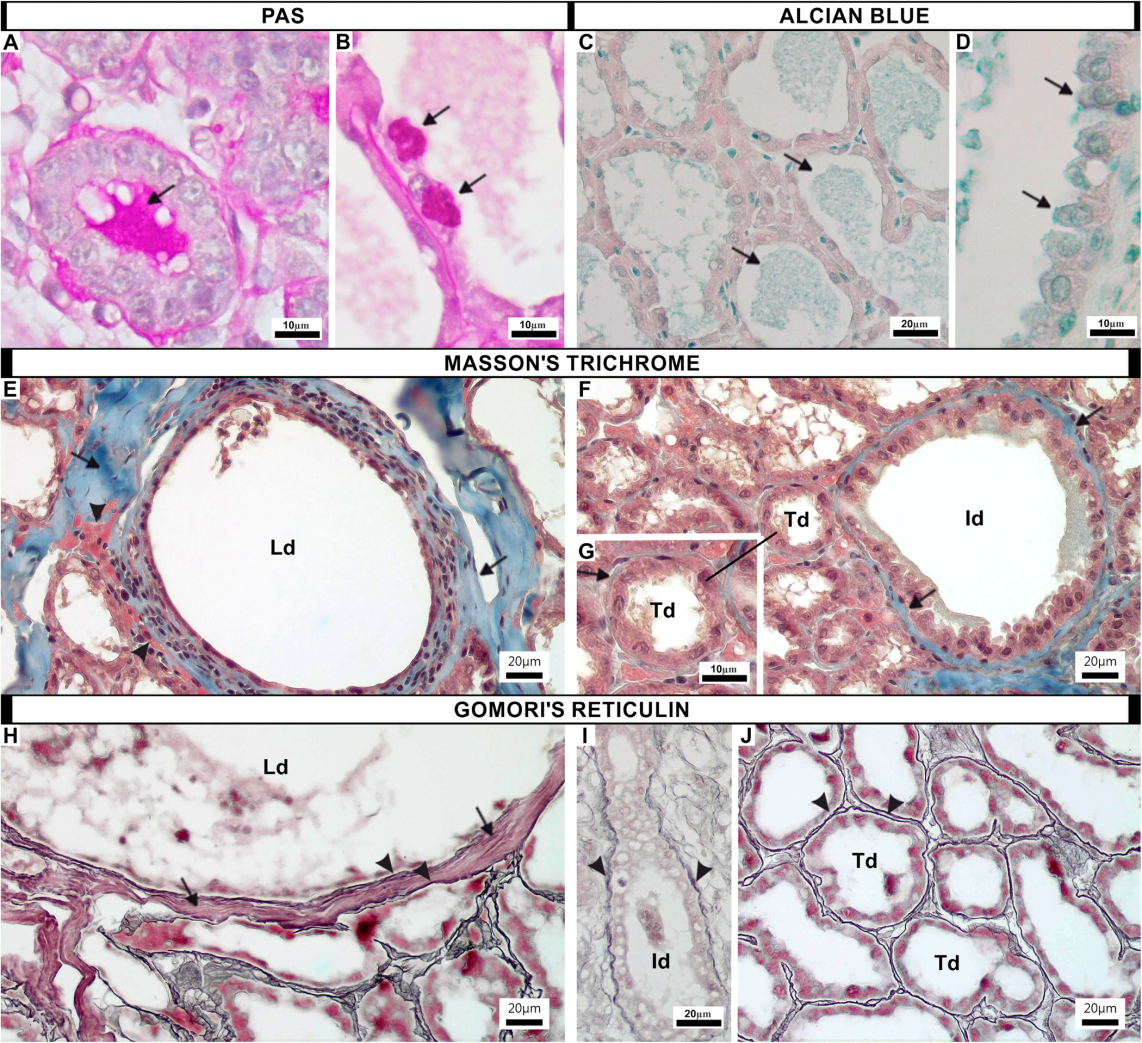
Histological sections of the mammary gland of LAC females of *Artibeus lituratus* stained with periodic acid‐Schiff (PAS, A, B), Alcian Blue (C, D), Masson's trichrome (E–G) and Gomori's reticulin (H–J). (A, B) Terminal ductules. Milk both in the lumen (A) and that secreted by secretory cells (B) present a large amount of PAS‐positive glycoproteins (arrows). (C, D) Terminal ductules. Milk both in the lumen (A) and that secreted by secretory cells (B) present acidic mucins (arrows). (E) Connective tissue surrounding the lactiferous duct. Greater quantity of collagen fibers (arrows) and few muscle fibers (arrowheads). (F) Connective tissue surrounding the intermediate duct. Smaller amount of collagen fibers (arrows). (G) Connective tissue surrounding the terminal ductules. Thin layer of collagen fibers (arrow). (H) Connective tissue surrounding the lactiferous duct. Highly intricate network of reticular fibers (arrowheads) and the greater quantity of type I collagen fibers (arrows) surrounding the lactiferous duct. (I) Connective tissue surrounding the intermediate duct. Highly intricate network of reticular fibers (arrowheads) surrounding the intermediate duct. (J) Connective tissue surrounding the terminal ductules. Thin and highly intricate network of reticular fibers (arrowheads) surrounding the terminal ductules. (Id, intermediate duct; Ld, lactiferous duct; Td, terminal ductules).

Similarly to other mammals, milk secreted into the alveolar lumen of *A. lituratus* may present polymorphonuclear neutrophils (Figure 2I). The milk also showed large amounts of PAS‐positive glycoproteins (Figure 3A,B) and acidic mucins (Figure 3C,D), both produced and released by the secretory cells.

The stroma of the mammary glands of *A. lituratus* varied in thickness, with a thick layer of collagen fibers around the lactiferous (Figure 3E) and intermediate ducts (Figure 3F), and a much thinner layer between the terminal ductules (Figure 3F,G). Gomori's reticulin staining revealed a highly intricate network of reticular fibers around all ductal structures of the mammary glands, including the lactiferous (Figure 3H), intermediate ducts (Figure 3I) and terminal ductules (Figure 3J). In contrast, type I collagen fibers were more abundant only around the lactiferous ducts (Figure 3H).

**Figure 4:**
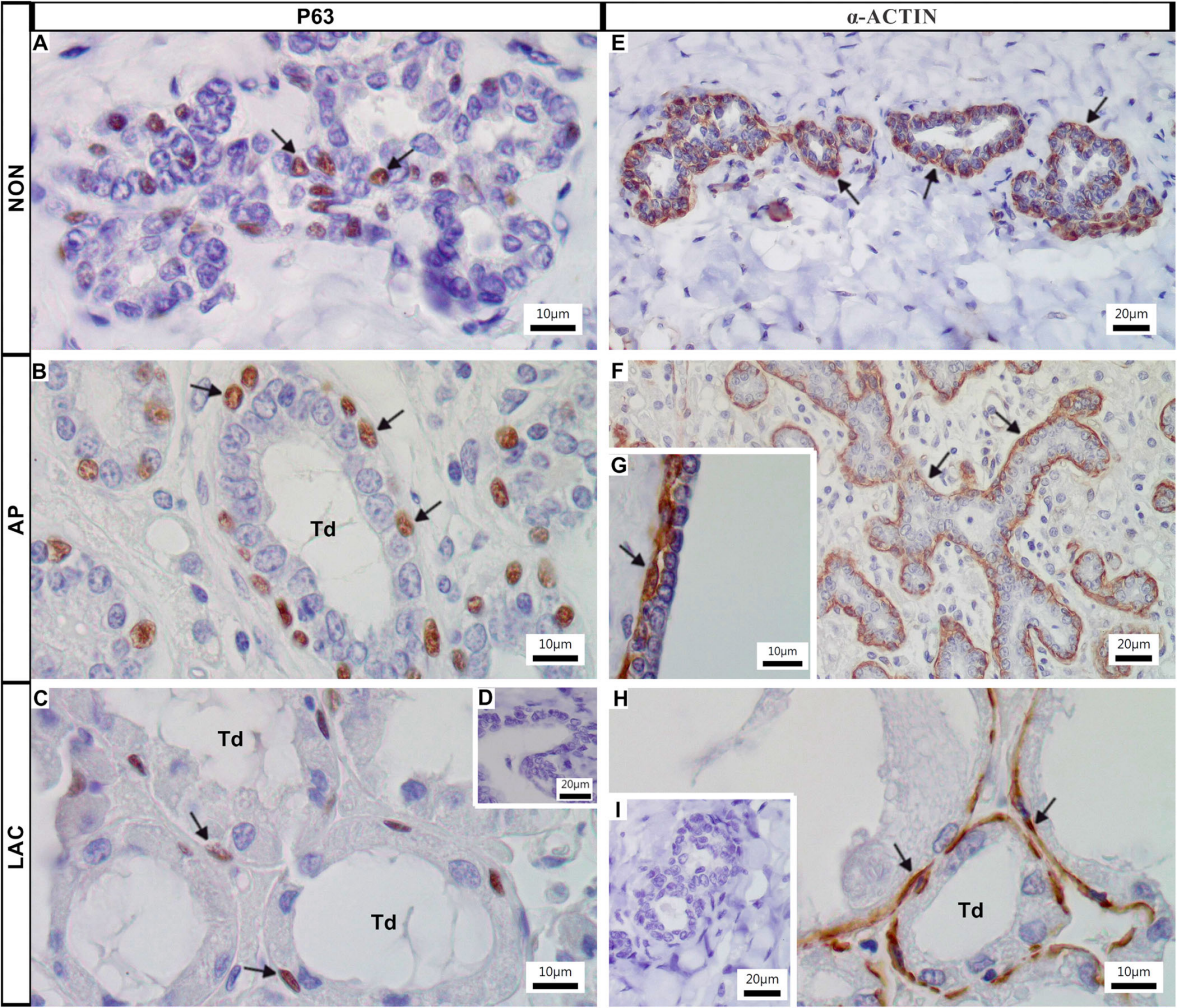
Histological sections of the mammary gland of *Artibeus lituratus* submitted to immunoreaction for the p63 (A–D) and for α‐actin (E–I). (A, E) NON females. (B, F–G) AP females. (C, H) LAC females. (D, I) Negative controls. Immunohistochemical reaction to the basal cell marker (p63) confirmed the existence of basal cells in epithelium of terminal ductules (arrows, A–C). Immunoreaction for α‐actin demonstrates that myoepithelial cells form a continuous outer layer around the epithelium (arrows, E–H).

Immunohistochemistry for p63 confirmed the presence of basal cells in the epithelium of the ducts of all analyzed groups (Figure 4A–C); while myoepithelial cells did not express this marker. Figure 4D shows the negative control for the p63 immunoreaction. Immunohistochemistry for α‐actin showed that myoepithelial cells formed a continuous outer layer surrounding the epithelium of the ducts of all analyzed groups (Figure 4E–H), while basal cells did not express this marker. Figure 4I shows the negative control for the α‐actin immunoreaction.

### 3.3 Development, Branching, and Involution

**Figure 5:**
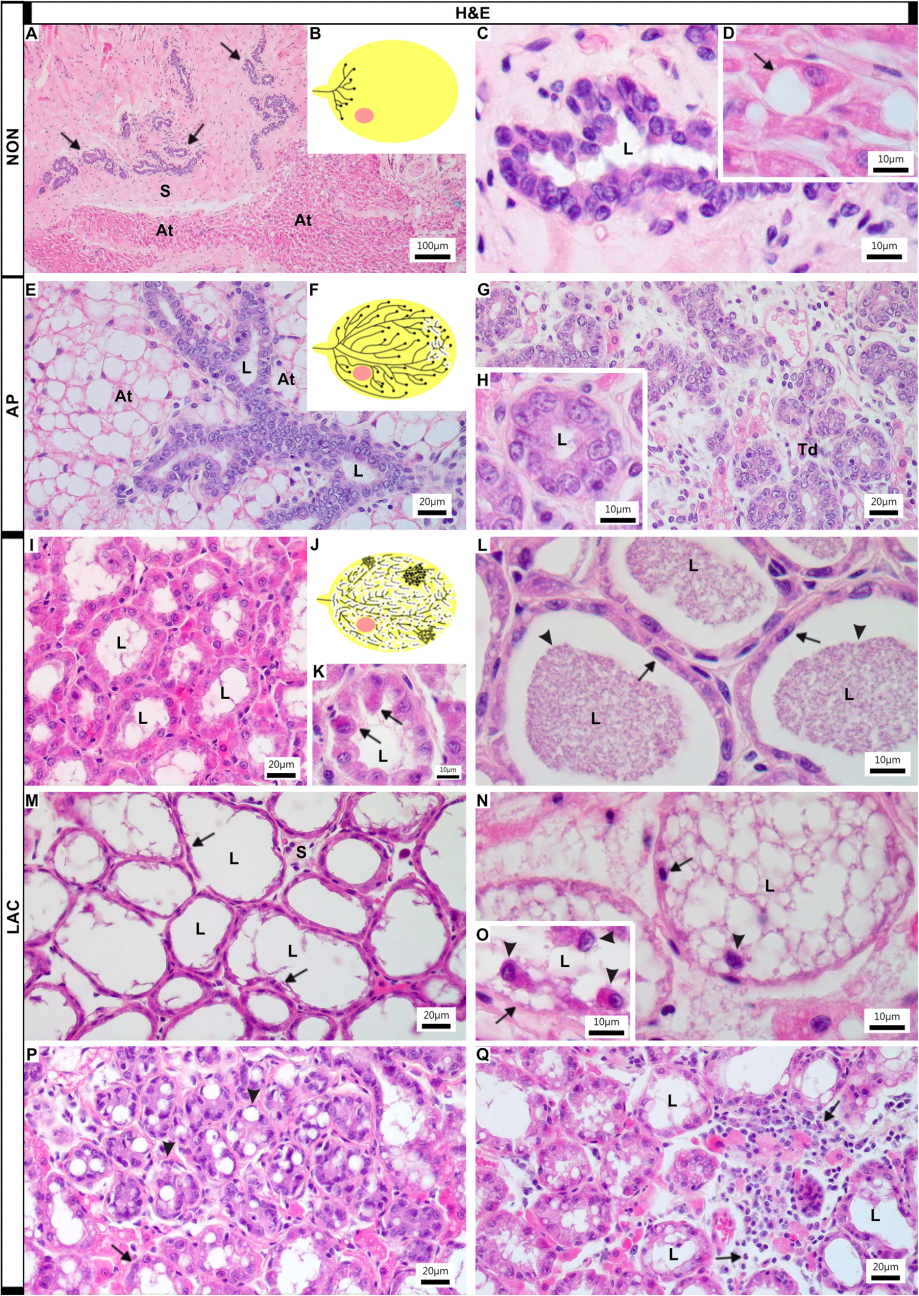
Histological sections of the mammary gland of *Artibeus lituratus* stained with hematoxylin and eosin. (A–D) NON females. Mammary gland is poorly developed, being composed of few ducts (arrows, A) interspersed within a rich stroma and covered by a large amount of adipose tissue (A, B). The ducts are lined by a poorly organized epithelium (C) and the adipocytes have a more elongated shape, but with an initial accumulation of lipids (arrow, D). (B) is a schematic drawing representing the little branching of the glandular epithelium in the adipose tissue of NON females. (E–H.) AP females. The mammary gland presents a more developed morphology, with ducts that branch in all directions through the adipose tissue (E, F). The beginning of ductal differentiation, with some terminal ductules differentiating into alveoli, which is composed of small and still barely evident lumen (G, H). (F) is a schematic drawing representing the large number of branches of the ducts and the formation of the first alveoli in AP females. (I–Q). LAC females. LAC females present up to five different types of alveolar morphophysiologies: the first formed by large differentiate alveoli (I), with turgid cells, which already secrete into the lumen (arrows, K); the second is characterized by large alveoli with uniform and well‐structured epithelium (arrows) and wide lumen, which contains milk (arrowheads, L); the third has flattened cells and rare foci of secretion (arrows, M); the fourth is composed of a disorganized epithelium, which presents some focus of cell death, with the detachment and release of epithelial cells into the lumen (arrowheads, N, O). Some alveoli did not even retain their cell lining (arrows, N, O); the last corresponds to new areas of alveolar branching and development (arrowheads, P) that occurs on the periphery of other already fully developed alveoli (arrow, P). The mammary gland of *A. lituratus* has a large number of inflammation/infiltration foci (arrow, Q). (J) is a schematic drawing demonstrating the predominance of secretory alveoli, some regions with loose epithelial cells in the lumen, at the same time as new regions of branching in the mammary gland of LAC females. (At, adipose tissue; L, lumen; S, stroma; Td, terminal ductules). In the schematic drawings, the yellow circle represents adipose tissue, the black lines represent glandular branches and the pink circle represents the associated axillary lymph node.

The mammary gland of NON females was poorly developed, with only a few ducts located near the nipple. These ducts exhibited few branches, which were interspersed within a rich stroma, composed of a broad layer of connective tissue and covered by a large amount of adipose tissue (Figure 5A,B). The ducts were lined by a poorly organized epithelium (Figure 5C). The adipocytes had a more elongated shape, with large and rounded nuclei; however, many cells already showed initial signs of lipid accumulation, becoming more rounded in shape (Figure 5D).

The mammary gland of AP females presented a more developed morphology, with ducts that branched through fully developed adipose tissue (Figure 5E,F). Differentiation into lactiferous and intermediate ducts, and terminal ductules was evident, as well as alveolar differentiation in a few regions of the gland (Figure 5G). However, these alveoli remained small, with poorly defined lumens (Figure 5G,H).

The mammary gland of LAC females showed marked morphological variability, even within the same gland, presenting up to five distinct alveolar morphophysiological types identified (Figure 5J). The first type included differentiated alveoli with turgid cells (Figure 5I), some showing an accumulation of intracytoplasmic secretions, while others already secreted into the lumen (Figure 5K). The second type was characterized by larger mature secretory alveoli with well‐structured epithelium and wide lumen, which contained milk secretion (Figure 5L). The third type presented an accentuated decrease in the height of the secretory epithelium, presenting flattened cells and rare foci of milk secretion (Figure 5M). The fourth type comprised a disorganized epithelium, which presented some foci of cell death, with the detachment and release of epithelial cells into the lumen (Figure 5N,O). Some alveoli did not even retain their epithelial cell lining (Figure 5N,O). The last type corresponded to areas of alveolar branching and development that occurred on the periphery of other already fully developed alveoli. These new alveoli presented epithelium that was still being organized and a barely evident lumen (Figure 5P).

The mammary glands of all *A. lituratus* groups presented a large number of foci of infiltrated blood cells (Figure 5Q), which could be seen dispersed in the stroma (Figure 5Q), in the lumen with milk (Figure 2K), or associated with the lactiferous ducts.

### 3.4 Stereology

**Figure 6:**
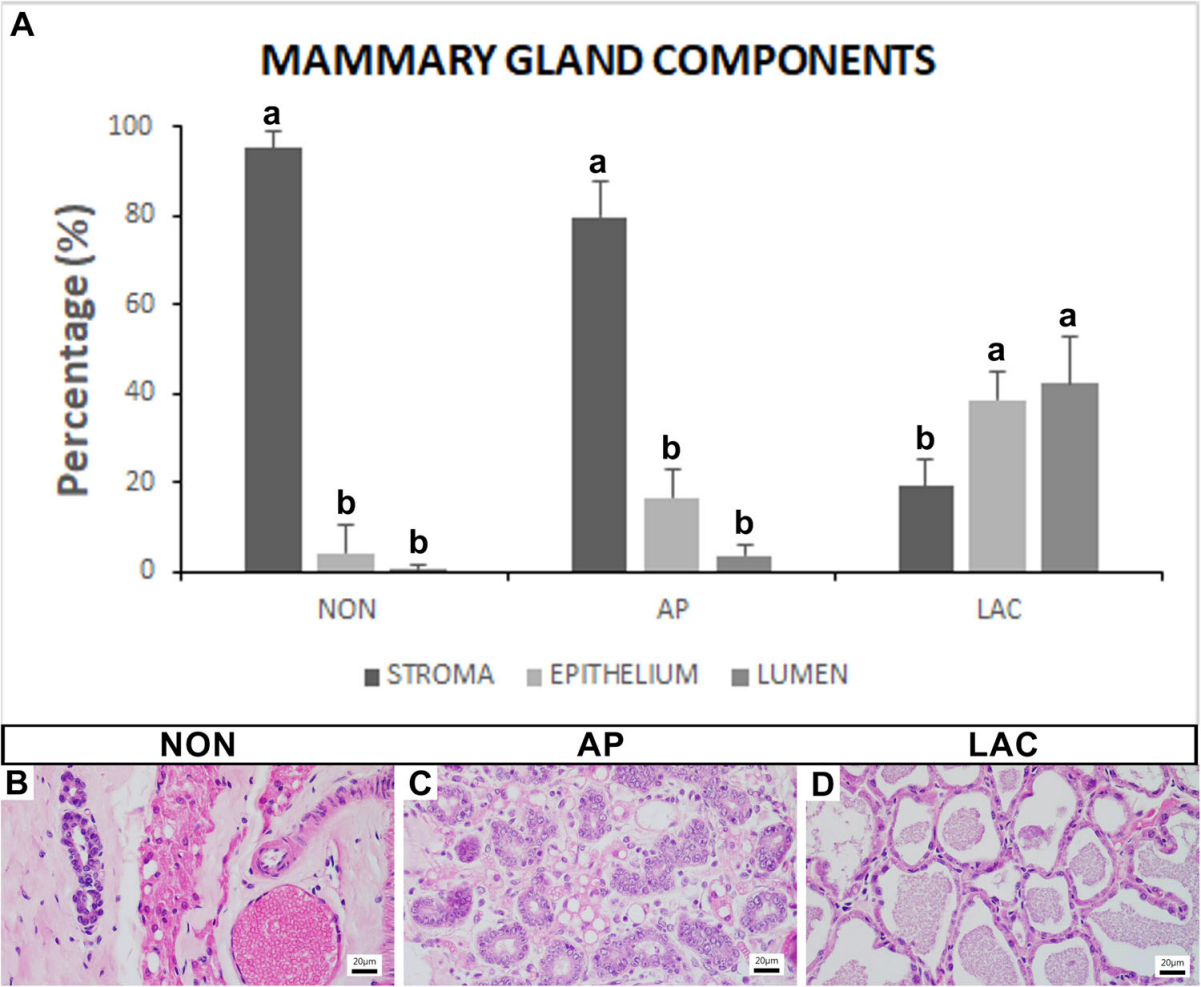
Stereology (amount of epithelium, lumen and stroma) of the mammary glands of *Artibeus lituratus*. (A) Graph showing the stereology (amount of epithelium, lumen and stroma). Data were expressed as means and standard deviations. Different letters indicate statistically significant differences (Kruskal‐Wallis at *p* ≤ 0.05; *n* = 5). (B) Histological section of the mammary gland of a NON female stained with hematoxylin and eosin. (C) Histological section of the mammary gland of an AP female stained with hematoxylin and eosin. (D) Histological section of the mammary gland of a LAC female stained with hematoxylin and eosin.

The stereology of the mammary glands of NON and AP females revealed similar results (Figure 6A,C). In both groups, the stroma was predominant (NON: 95.42 ± 3.5%; AP: 79.92 ± 7.76%), but the epithelium (NON: 3.99 ± 6.5%; AP: 16.59 ± 6.1%) and lumen were not abundant (NON: 0.58 ± 1.1%; AP: 3.47 ± 2.34%). However, when the amount of epithelium and lumen were combined in these groups (NON = 4.57; AP = 20.06) — representing the functional portion of the gland — the values quadrupled in the AP females, which indicates substantial tissue remodeling and stimulation/preparation for lactation. Conversely, in LAC females (Figure 6D) there was a significant decrease in the amount of stroma (19.18 ± 5.72%) and concomitant significant increases in epithelium (38.67 ± 5.98%) and lumen (42.14 ± 10.94%).

### 3.5 Immunohistochemistry

#### 3.5.1 Estrogen Receptor α (ERα)

**Figure 7:**
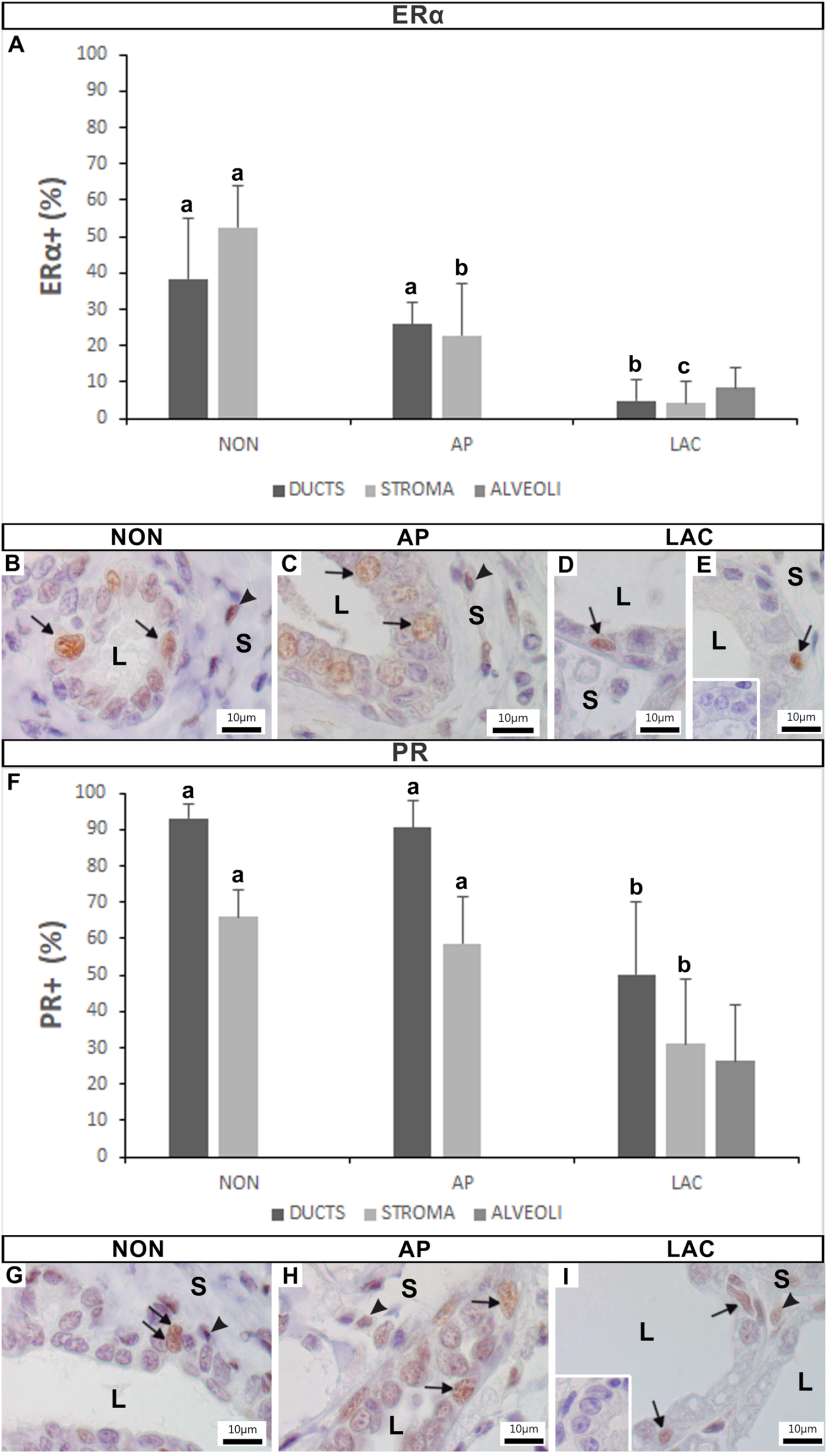
Variation in the percentage of ERα (A–E) and PR (F–I) positive cells in the mammary gland of *Artibeus lituratus* in the different analyzed groups. (A) Graph showing the percentage of ERα+ cells. (B) NON female. (C) AP female. (D,E) LAC females. The marking in the epithelial (arrows) and stromal (arrowheads) cells. Inset in E is the negative control of the ERα immunoreaction. (F) Graph showing the percentage of PR+ cells. (G) NON female. (H) AP female. (I) LAC female. The marking in the epithelial (arrows) and stromal (arrowheads) cells. Inset in I is the negative control of the PR immunoreaction. Data were expressed as means and standard deviations. Different letters indicate statistically significant differences (Kruskal‐Wallis at *p* ≤ 0.05; *n* = 5). (L, lumen; S, stroma).

The epithelial (secretory and basal cells) and stromal cells of the mammary gland of *A. lituratus* exhibited ERα immunoreactivity (Figure 7A–E). The reactivity varied significantly among the analyzed groups (Figure 7A). The epithelium of the ducts of NON (Figure 7B) and AP females (Figure 7C) presented respectively 38.43 ± 16.75% and 25.78 ± 6.29% ERα+ cells (Figure 7A), with no significant difference among these groups. However, LAC females (Figure 7D) displayed significantly fewer ERα+ cells in the epithelium ducts, with 8.5 ± 5.54%. Conversely, the stroma of the mammary glands of NON females (Figure 7A) presented a significantly higher percentage of ERα+ cells (52.42 ± 11.43%) compared with the AP (22.91 ± 14.04%) and LAC females (4.25 ± 5.71%). In the LAC females, only 8.5 ± 5.55% of the epithelial cells in the alveoli expressed ERα (Figure 7A and E). The inset in Figure 7E shows the negative control for the ERα immunoreaction.

#### 3.5.2 Progesterone Receptor (PR)

The epithelial (secretory and basal cells) and stromal cells of the mammary gland of *A. lituratus* showed PR immunoreactivity (Figure 7F–I). There were significant variations among the analyzed groups (Figure 7F). In the epithelium of the ducts, NON (Figure 7G) and AP females (Figure 7H) presented 93.17 ± 4.02% and 90.44 ± 7.68% PR+ cells (Figure 7F) respectively, with no significant difference between the groups. However, LAC females presented significantly fewer PR+ cells (50.1 ± 19.95%). Similarly, the stroma of the mammary glands of the NON (Figure 7G) and AP (Figure 7H) females showed similar percentage of PR+ cells, 66.02 ± 7.48% and 58.66 ± 12.75% PR+ cells (Figure 7F), respectively, but with a significant decrease in the immunoreactivity in the stroma of LAC females, with 31.12 ± 17.75% PR+ cells (Figure 7I). In turn, the alveoli of LAC females presented PR+ cells in only 26.35 ± 15.33% of their epithelial cells (Figure 7F and I). The inset in Figure 7I represents the negative control for the PR immunoreaction.

#### 3.5.3 Proliferating Cell Nuclear Antigen (PCNA)

**Figure 8:**
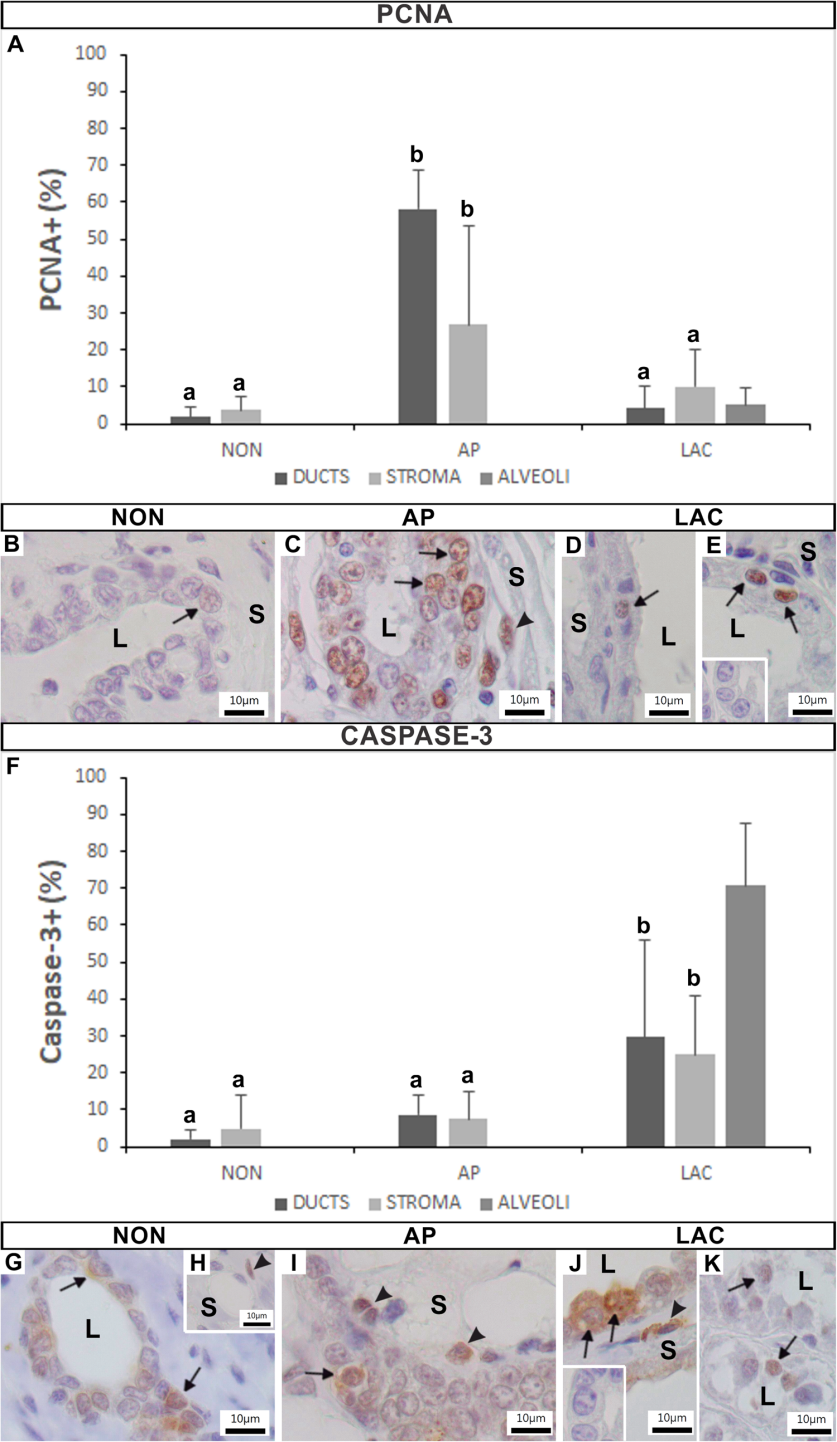
Variation in the percentage of PCNA (A–E) and Caspase‐3 (F–K) positive cells in the mammary gland of *Artibeus lituratus* in the different analyzed groups. (A) Graph showing the percentage of PCNA+ cells. (B) NON female. (C) AP female. (D, E) LAC females. The marking in the epithelial (arrows) and stromal (arrowheads) cells. Inset in E is the negative control of the PCNA immunoreaction. (F) Graph showing the percentage of Caspase‐3+ cells. (G, H) NON females. (I) AP female. (J, K) LAC females. The marking in the epithelial (arrows) and stromal (arrowheads) cells. Inset in J is the negative control of the Caspase‐3 immunoreaction. Data were expressed as means and standard deviations. Different letters indicate statistically significant differences (Kruskal‐Wallis at *p* ≤ 0.05; *n* = 5). (L, lumen; S, stroma).

The epithelial (secretory and basal cells) and stromal cells of the mammary gland of *A. lituratus* presented PCNA immunoreactivity (Figure 8A–E). The epithelium of the ducts of the NON (Figure 8B) and LAC females (Figure 8D) had similar percentage of PCNA+ cells, showing 1.71 ± 2.74% and 4.17 ± 5.96%, respectively (Figure 8A). However, AP females presented significantly more PCNA+ cells, with 58.12 ± 10.81% (Figure 8C). Similarly, the stroma of the mammary glands of NON (Figure 8B) and LAC (Figure 8D,E) females exhibited similar percentage of PCNA+ cells, with 3.63 ± 3.86% and 10.05 ± 11.75%, respectively (Figure 8A), but there was a significant increase in the stroma of AP females, with 26.87 ± 23.75% PCNA+ cells (Figure 8C). In turn, the alveoli of LAC females presented PCNA in only 5.06 ± 4.61% of their epithelial cells (Figure 8A and E). The inset in Figure 8E represents the negative control for the PCNA immunoreaction.

#### 3.5.4 Activated Caspase‐3 (Caspase‐3)

The results showed that both epithelial (secretory and basal cells) and stromal cells of the mammary gland of *A. lituratus* exhibited Caspase‐3 immunoreactivity (Figure 8F–K). There was significant variation among the analyzed groups (Figure 8F). The epithelium of the ducts of NON (Figure 8G) and AP females (Figure 8I) presented similar percentage of Caspase‐3+ cells, showing 2.15 ± 2.41% and 8.53 ± 5.54% cells, respectively (Figure 8F). However, there was a significant increase in the ducts of LAC females, with 29.68 ± 26.24% Caspase‐3+ cells (Figure 8F and J). Similarly, the stroma of the mammary glands of NON (Figure 8H) and AP (Figure 8I) females presented similar values, 4.86 ± 8.99% and 7.44 ± 7.35% Caspase‐3+ cells, respectively (Figure 8F), but there was a significant increase in the stroma of LAC females, with 24.96 ± 15.85% Caspase‐3+ cells (Figure 8J). In turn, the alveoli of LAC females present the highest percentage, 70.8 ± 16.54% of their epithelial cells expressed this protein (Figure 8A and E). The inset in Figure 8J represents the negative control for the Caspase‐3 immunoreaction.

## 4 Discussion

### 4.1 Morphology and Development

The mammary gland of adult NON females of the bat *A. lituratus* consists of a rudimentary, quiescent tree of ducts, surrounded by abundant unilocular adipose tissue and fibrous stroma. This pattern is similar to that observed in virgin females of the bat *Carollia perspicillata* (Evarts et al. 2004) and in non‐lactating females of other mammals, such as *Nasua nasua* (Casals et al. 2013) and *Meriones unguiculatus* (Leonel et al. 2017). In *A. lituratus*, the rudimentary lactiferous ducts exhibited few branches concentrated in a single region of the axillary mammary gland. This axillary positioning appears to be an exclusive characteristic of certain bat species (Evarts et al. 2004; Cardiff and Allison 2012; Beguelini et al. 2020).

During pregnancy, the mammary gland undergoes progressive ductal branching driven by epithelial cell proliferation, accompanied by a reduction in adipose tissue. At the end of pregnancy, alveolar differentiation begins, marked by presecretory activity where epithelial cells synthesize and store milk components, such as protein, lipids, and lactose (Nandi 1958; Cardiff et al. 2018). Similarly, the lactiferous ducts of AP females of *A. lituratus* show increased branching and initial alveolar formation. However, these alveoli remain small and poorly developed, presenting a barely evident lumen without milk. This pattern differs from that reported for the bat *Myotis nigricans* (Beguelini et al. 2020) and the rodent *Mus musculus* (Richert et al. 2000), in which alveoli are already well developed and filled with milk by the end of gestation.

The beginning of the lactation is characterized by intense secretory activity (Evarts et al. 2004; Cardiff et al. 2018). The newborn's sucking stimulus induces the contraction of myoepithelial cells and release of the milk from the alveoli and ducts. This continuous stimulation maintains the functionality of the gland (Richert et al. 2000; Neville 2001; Gjorevski and Nelson 2011; Sriraman 2017). Similarly to most mammals (Evarts et al. 2004; Bellatine et al. 2010; Casals et al. 2013), LAC females of *A. lituratus* exhibit mammary glands rich in milk‐secreting alveoli, sparse stroma, and minimal adipose tissue confined to the periphery of the gland. This architecture resembles that of *C. perspicillata* (Evarts et al. 2004) and *M. nigricans* (Beguelini et al. 2020), and *M. unguiculatus* (Leonel et al. 2017), but differs from mice, where few alveoli are surrounded by abundant adipose tissue (Evarts et al. 2004).

Despite these similarities, the mammary gland of LAC females of *A. lituratus* is not a completely homogeneous organ. The presence of newly branching areas (indicative of alveolar differentiation) alongside regions of tissue degeneration suggests ongoing remodeling during lactation. This continuous renewal may sustain milk production throughout the lactation period.

Proper mammary development and functional differentiation into milk‐producing organs are totally dependent on epithelial‐stromal interactions (Robinson et al. 1999). The mammary stroma contains different cell types, such as adipocytes, fibroblasts, and leukocytes — many of which produce factors that modulate epithelial proliferation and differentiation (Gouon‐Evans et al. 2000). Leukocyte recruitment and infiltration to the stroma is essential for ductal branching and gland remodeling, assisting epithelial expansion into the surrounding adipose tissue (Gouon‐Evans et al. 2000). During lactation, leukocytes also respond to invasive pathogens, inducing local inflammation to eliminate intruders (Unsworth et al. 2014), while during involution, macrophages facilitate epithelial cell clearance and mammary remodeling (O'Brien et al. 2012).

In *A. lituratus*, several inflammatory foci were observed throughout the mammary stroma. In AP females, leukocytes were predominantly located around the lactiferous ducts, possibly assisting with ductal branching (Gouon‐Evans et al. 2000). In LAC females, leukocytes are dispersed in the stroma around the alveoli or grouped near the lactiferous ducts. Since bat pups remain attached to the nipple during flight (Evarts et al. 2004), the mechanical stress exerted by their weight and movement may cause micro‐injuries to the ducts, potentially explaining the inflammatory response in these regions. Additionally, the high number of inflammatory foci in LAC females may be related to glandular remodeling, enabling the formation of new alveoli and the replacement of areas undergoing epithelial cell death (Gouon‐Evans et al. 2000).

Similarly to most mammals, including *Homo sapiens* and *M. musculus* (Lazard et al. 1993; Hassiotou and Geddes 2013; Cardiff et al. 2018), α‐actin immunoreactivity revealed a continuous outer layer of myoepithelial cells surrounding the ducts and alveoli in all analyzed groups of *A. lituratus*. However, unlike studies that define the mammary epithelium as a bilayer consisting of outer myoepithelial cells and inner luminal cells (Hitchcock et al. 2020), p63 immunostaining demonstrated the presence of basal cells in *A. lituratus*. This organization resembles the structure proposed for ruminants (Rainard et al. 2022).

The mammary gland of *A. lituratus* also contains a large amount of collagen around the lactiferous ducts, the blood vessels, and in some bands scattered in the mammary stroma. This pattern closely resembles that of *C. perspicillata* (Evarts et al. 2004). The abundance of collagen in the mammary gland of bats may provide structural reinforcement, allowing the gland to support the mechanical stress of carrying a pup during flight (Evarts et al. 2004).

### 4.2 Hormonal Control: Emphasis on Estradiol and Progesterone

Hormonal control of mammary gland development involves a complex interplay of multiple hormones to regulate different stages of glandular growth and function (Brisken and O'malley 2010). It is generally accepted that all prepubertal development of the mammary gland is largely hormone independent (Brisken and Ataca 2015). However, with the onset of puberty, and during pregnancy and lactation, mammary gland development becomes strictly hormone‐dependent (Brisken and O'malley 2010; Brisken and Ataca 2015). In adult females, estradiol and progesterone, in combination with prolactin and growth hormone (GH), are required to drive ductal elongation, side branching, and alveolar differentiation (Brisken and O'malley 2010; Brisken and Ataca 2015).

It is widely recognized that estradiol or different estrogen metabolites, acting via ERα, is required for ductal elongation. ERα+ cells mediate estrogenic stimulation by releasing paracrine factors that promote proliferation of adjacent cells, thereby driving ductal elongation (Bocchinfuso and Korach 1997; Mueller et al. 2002; Mallepell et al. 2006). Progesterone, on the other hand, signals through the PR to promote ductal side branching and alveolar differentiation (Brisken et al. 1999; Brisken and O'malley 2010; Brisken and Ataca 2015).

It is important to highlight that the present study was conducted using the same animals previously analyzed by Santiago et al. (2020), thus sharing identical serum hormone profiles. The average serum estradiol concentration was 103.41 ± 68.23 pg/mL in NON females, 6069.76 ± 600 pg/mL in AP females, and 263.15 ± 105.03 pg/mL in LAC females, with significantly higher levels in AP females compared to NON and LAC groups. Progesterone levels followed a similar pattern: 14.78 ± 8.66 ng/mL in NON females, 41.93 ± 23.73 ng/mL in AP females, and 8.31 ± 2.65 ng/mL in LAC females, with AP females again showing significantly higher values.

Immunohistochemistry revealed that NON females of *A. lituratus* had the highest percentage of ERα+ cells in both the epithelium and stroma, despite having a lower circulating estradiol level. This high receptor marking may reflect the retention of ERα synthesized during puberty, awaiting estradiol stimulation during early pregnancy to trigger proliferation and ductal elongation (Kenney et al. 2003). Notably, estradiol serum levels in NON females of *A. lituratus* were comparable to those observed during the estrous cycle of *Rattus norvegicus* (Fata et al. 2001).

During pregnancy, estradiol continues to be produced by the ovaries, with additional contribution from the placenta (Gibori et al. 1988). Estradiol levels peak during late pregnancy, and this hormone indirectly induces mammary gland development and differentiation (Brisken and Ataca 2015). In *A. lituratus*, AP females show higher serum estradiol levels than those reported in pregnant *R. norvegicus*, *M. musculus*, and *H. sapiens* (Loriaux et al. 1972; McCormack and Greenwald 1974; Rosenblatt 1988). However, despite the highest estradiol serum levels, ERα marking in the epithelium and especially in the stroma declines in AP females, possibly due to negative feedback regulation (compensation) by high circulating estradiol levels, which can counterbalance and regulate the accentuated ductal proliferation during this phase (Shyamala et al. 2002) that also coincides with the peak of proliferative activity (PCNA). Other mammals such as *R. norvegicus* and *M. musculus* do not show a decrease in ERα in the epithelium during pregnancy (Shyamala et al. 1992; Saji et al. 2000). In contrast, LAC females exhibit both lower circulating estradiol concentration and reduced ERα. Nevertheless, the presence of newly branching regions in LAC females suggests that even low estradiol levels may be sufficient to activate the remaining ERα+ cells in the epithelium and stroma to support ongoing alveolar development during this phase.

*Artibeus lituratus* shows a high number of ERα+ stromal cells, especially in NON and AP females, and a low number in LAC females. These findings, along with the high circulating estradiol level in AP females and the low level in LAC females, seem to explain the highest ductal proliferative activity (PCNA) in AP females. This pattern supports the idea that ERα+ stromal cells respond to estradiol by producing mitogenic factors that act on the epithelium (Cunha et al. 1997). Interestingly, this differs from what has been described for *M. musculus*, where ERα+ stromal cells are limited to areas near the nipple (Shyamala et al. 2002).

While estradiol via ERα stimulates ductal elongation, progesterone via PR regulates side branching and alveologenesis (Brisken et al. 1999; Arendt and Kuperwasser 2015). PR is a nuclear receptor that responds to progesterone stimuli (Evans 1988). Its expression is induced by estradiol through ERα activation (Schultz et al. 2003). Once activated, PR drives side branching, the formation of intermediate ducts and, together with prolactin, promotes terminal duct differentiation into secretory alveoli (Brisken et al. 1999; Brisken 2002).

Although NON females of *A. lituratus* exhibit low progesterone levels—comparable to levels during the estrous cycle in *R. norvegicus* (Fata et al. 2001)—they show high PR marking. This may be attributed to the high number of ERα+ cells in this group, as estrogen stimulation is known to upregulate PR expression (Schultz et al. 2003; Petz et al. 2004). Despite high PR marking, proliferative activity remains low (low PCNA), suggesting that both estrogen and progesterone are necessary to initiate branching morphogenesis. Additionally, the increase in progesterone stimuli, along with prolactin expression, act to stimulate alveolar differentiation and milk production (Brisken et al. 1999). Interestingly, the level of PR marking in NON females of *A. lituratus* is comparable to that seen in *M. musculus* (Shyamala et al. 1997).

PR activation is crucial for side branching and alveologenesis (Brisken et al. 1998; Lydon et al. 1995). Accordingly, PR+ cells peaks in AP females of *A. lituratus*, the phase associated with the most extensive side branching and early alveolar formation. When compared to other mammals, the PR marking in *A. lituratus* closely resembles that of *R. norvegicus* and *M. musculus* (Shyamala et al. 2002). The high proliferative activity and side branching seen in AP females are likely driven by the combined effects of estradiol acting via ERα and progesterone via PR, which trigger extensive mitotic activity and side branching in this phase (Brisken et al. 1998; Lydon et al. 1995).

In LAC females of *A. lituratus*, once the mammary gland is fully differentiated and functional, both serum progesterone and PR marking decrease. This decrease likely reflects the gland's advanced differentiation status (Shyamala et al. 2002). However, residual PR marking may help sustain secretory activity and support new branching, as observed in this group. Despite lower PR marking, the proportion of PCNA+ cells indicates that the tissue remains proliferative.

Similar to ERα, PR is also expressed in the mammary stroma of all *A. lituratus* groups. The pattern of PR marking in the stroma closely mirrors that of the epithelium, suggesting that PR+ stromal cells stimulated by progesterone possibly act through paracrine mechanisms in epithelial cells. This distinguishes *A. lituratus* from *M. musculus* and *H. sapiens*, which lack stromal PR marking (Press and Greene 1988; Shyamala et al. 1997).

Regarding proliferative activity (based on PCNA), the mammary gland of *A. lituratus* shows a significant increase only during pregnancy (AP females), likely driven by the synergistic action of high estradiol and progesterone levels. In contrast, during lactation (LAC females), a significant increase in Caspase‐3 marking was observed, particularly in the ducts and alveoli. Caspase‐3 is a key effector protease in the apoptotic cascade and is considered a hallmark of programmed cell death in epithelial tissues (Porter and Jänicke 1999). Its activation in the mammary gland typically reflects the onset of alveolar remodeling, regression, or turnover of secretory epithelial cells, which may occur even during the lactation phase (Watson 2006). This may reflect the continuous apocrine secretion process, which eventually leads to epithelial cell death and alveolar remodeling. To maintain lactation, the gland of *A. lituratus* likely undergoes cycles of alveolar renewal and ductal branching, consistent with the observed coexistence of proliferative and degenerative regions in the mammary tissue of LAC females.

## 5 Conclusion

The mammary gland of *A. lituratus* exhibits a similar pattern to that of other mammals. However, there are some notable differences, including an epithelial organization composed of epithelial, basal, and myoepithelial cells, as well as evidence of continuous remodeling, branching, and renewal during lactation, with accentuated epithelial cell death. Glandular development and branching during advanced pregnancy seem to be stimulated by a significant increase in circulating estradiol, which, via ERα activation, stimulates ductal proliferation, and by a concurrent increase in progesterone, which, through PR activation and in synergy with prolactin, stimulates side branching and alveolar differentiation.

## Authors Contribution

**Cornélio S. Santiago:** conceptualization, data curation, formal analysis, investigation, roles/writing – original draft. **Pollyana B. Pimentel:** data curation, formal analysis, investigation. **Emília M. Soares:** data curation, formal analysis, investigation. **Juliana F. Ferraz:** data curation, formal analysis, investigation. **Luiz H. A. Guerra:** data curation, formal analysis, investigation. **Carolina C. Souza:** data curation, formal analysis, investigation. **Rejane M. Góes:** supervision, writing – review and editing. **Eliana Morielle‐Versute:** supervision, writing – review and editing. **Sebastião R. Taboga:** supervision, writing – review and editing. **Mateus R. Beguelini:** conceptualization, data curation, formal analysis, funding acquisition, investigation, methodology, project administration, resources, roles/writing – original draft.

## Ethics Statement

The captures were authorized by the Brazilian Institute of Environment (Instituto Brasileiro do Meio Ambiente, IBAMA – Process: 21707‐1). The experimental procedures were conducted according to the National Institutes of Health Guidelines for the Use of Laboratory Animals, and previously approved by the animal experimentation ethics committee of the São Paulo State University (CEUA–UNESP, Process: 013/09). Access to Genetic Legacy was approved by the Genetic Legacy Management Council of the Brazilian Ministry of the Environment (Process: AA38F12).

## Conflicts of Interest

The authors declare no conflicts of interest.

## Data Availability

All data generated or analyzed during this study will be available on request.
